# Cat-CrNP as new material with catalytic properties for 2-chloro-2-propen-1-ol and ethylene oligomerizations

**DOI:** 10.1038/s41598-021-94056-0

**Published:** 2021-07-26

**Authors:** Jacek Malinowski, Dagmara Jacewicz, Artur Sikorski, Mariusz Urbaniak, Przemysław Rybiński, Patrycja Parnicka, Adriana Zaleska-Medynska, Barbara Gawdzik, Joanna Drzeżdżon

**Affiliations:** 1 Faculty of Chemistry, University of Gdansk, Wita Stwosza 63, 80-308 Gdansk, Poland; 2 Institute of Chemistry, Jan Kochanowski University, Swietokrzyska 15 G, 25-406 Kielce, Poland

**Keywords:** Chemistry, Materials science

## Abstract

The contemporary search for new catalysts for olefin oligomerization and polymerization is based on the study of coordinating compounds and/or organometallic compounds as post-metallocene catalysts. However known catalysts are suffered by many flaws, among others unsatisfactory activity, requirement of high pressure or instability at high temperatures. In this paper, we present a new catalyst i.e. the crystalline complex compound possesing high catalytic activity in the oligomerization of olefins, such as 2-chloro-2-propen-1-ol and ethylene under very mild conditions (room temperature, 0.12 bar for ethylene oligomerization, atmospheric pressure for 2-chloro-2-propen-1-ol oligomerization). New material—Cat-CrNP ([nitrilotriacetato-1,10-phenanthroline]chromium(III) tetrahydrate) has been obtained as crystalline form of the nitrilotriacetate complex compound of chromium(III) with 1,10-phenanthroline and characterized in terms of its crystal structure by the XRD method and by multi-analytical investigations towards its physicochemical propeties The yield of catalytic oligomerization over Cat-CrNP reached to 213.92 g · mmol^−1^ · h^−1^· bar^−1^ and 3232 g · mmol^−1^ · h^−1^ · bar^−1^ for the 2-chloro-2-propen-1-ol and ethylene, respectively. Furthemore, the synthesis of Cat-CrNP is cheap, easy to perform and solvents used during preparation are environmentally friendly.

## Introduction

Since the 1990s, when post-metallocene catalysts were invented, research into polymerization catalysts and material and polymer products became more popular and carried out with greater intensity^1,2^. Nowadays, new materials are sought to act as catalysts for olefin polymerization and oligomerization^3–5^.

Philips catalyst, created by depositing chromium(VI) on silica gel with an oxide, is one of the most commonly used catalysts for the polymerization of olefin, next to Ziegler–Natta catalysts^6^. For many years, Philips type catalysts have been modified in order to obtain polyolefins with the desired properties^6^. The modifications of the Philips catalyst are based on chromium(V) oxide, chromium(III) oxide and chromium(II) oxide. The Philips catalyst is also used in the nanocarrier which is alumina. The common element in between the modification of the Philips catalyst and the post-metallocene chromium(III) catalysts is noticeable. New post-metallocene catalysts are often activated by modified methylaluminoxane, which contains significant amounts of aluminum^6^. Chromium(III) complex compounds are known as highly active catalysts for olefin oligomerization and polymerization^6^. The chromium(III) cation can be stabilized by various multi-donor ligands such as, for example, N-pyrrolidine pendant donor or *N,N,N*-bis(imino)trihydroquinoline^7,8^. Chromium(III) complex with chloride and *N,N,N*-bis(imino)trihydroquinoline is used as precatalyst and it must be activated by modified methylaluminoxane (MMAO)^8^. The mentioned chromium(III) complex upon activation is used for the polymerization of ethylene at 80 °C degrees for 1 h. The product of the polymerization reaction using this catalytic system is polyethylene. One of the possible four-donor ligand which stabilizes metal ions is nitrilotriacetate anion. Amine nitrilotriacetate salts e.g. di-n-butylamine salt of nitrilotriacetic acid are used for removal copper catalyst from poly(2,6-dimethyl-1,4-phenylene oxide)^9^, however, according to our best knowledge, there is no reports on the catalytic properties of chromium(III) complex compounds with the nitrilotriacetate anion for oligomerization and polymerization. In addition, nitrilotriacetic acid (H_3_NTA) was used in the synthesis of polymers in such a way that nitrilotriacetate Ni(II) complex end-functionalized polystyrene was obtained^10^. The nitrilotriacetate polymer material has been developed and prepared for use in controlled bioconjugation^11–13^. The complexes of transition metal cations containing a nitrilotriacetate anion are used for the purification and detection of proteins, provided that they have been tagged with oligohistidine^14–16^.

Nitrilotriacetate complexes with Mg^2+^, Ca^2+^, Sr^2+^, Ba^2+^ are used as coordination polymers for self-templating porous carbon electrocatalysts^17–20^. Crystalline complex compounds containing nitrilotriacetate anion and cations: Mg^2+^, Ca^2+^, Sr^2+^, Ba^2+^ were pyrolyzed at 750 ˚C in Ar atmosphere. Inorganic particles formed during pyrolysis were tested. These particles are, for example, MgO, BaCO_3_ which are carbon-deposited. These investigations allow to understanding the role of metal ions: Mg^2+^, Ca^2+^, Sr^2+^, Ba^2+^ in the formation of morphological diversity of the carbons which constitute electrocatalysts for reduction of oxygen^21^.

The first post-metallocene catalysts were nickel(II) and palladium(II) complex compounds with diimine ligands, which catalyzed the reaction of polyethylene formation. Brookhart and co-workers were pioneers in the design and research of post-metallocene catalysts^22^. The literature shows that modifications of these catalysts are often researched and used in olefin polymerization and copolymerization^23^. The modification of the ligand structure and the optimization of the conditions of the polymerization and copolymerization process have a significant impact on the catalytic activity of these complexes. These complexes allow a controlled influence on the microstructure of polymer chains^23^. Currently designing new nickel(II) catalysts, attention is paid to the ring-deformation of ligands^24^. Literature reports that modified diimine complexes of palladium(II) found their application in the "living" polyglass, however, due to the attachment of only one chain to the metal, industrial application of these test results is very difficult^25^.

Pioneering research in the field of iron and cobalt compounds-based catalysts with bis (arylimino) pyridine ligands was carried out by an independent group of Brookhart and Gibson^26,27^. Iron and cobalt complexes are also used as catalysts for ethylene oligomerization and polymerization. The development of the research conducted this day indicates a large influence of the deformation of the ligand ring on the catalytic activity^28^.

Currently, homogeneous chromium(III) precatalysts used for the oligomerization of ethylene are known. Most often these are catalysts containing bidentate ligands of the N^P, N^O and N^N type, tridentate N^N^N, N^N^O, N^O^N type and tetrahedral ligands N^N^N^N type^29^. Chromium(III) complex compounds are characterized by moderate activity in ethylene oligomerization at the temperature of the experiment within the range of 35–75 °C^29^.

In this work, new material possessing catalytic properties for 2-chloro-2-propen-1-ol and ethylene oligomerizations was successfully synthesized. Cat-CrNP is newly obtained nitrilotriacetate complex compound of chromium(III) with 1,10-phenanthroline ([nitrilotriacetato-1,10-phenanthroline] chromium(III) tetrahydrate). The nitrilotriacetate chromium(III) complex has been fully characterized by methods: XRD, elemental analysis, IR, MALDI-TOF–MS, potentiometric and conductometric titrations, thermal analysis and scanning electron microscopy. For the first time Cat-CrNP was used as a precatalyst with methylaluminoxane (MMAO-12) as an activator for the oligomerization of 2-chloro-2-propen-1-ol and ethylene.

## Results and discussion

**Figure 1:**
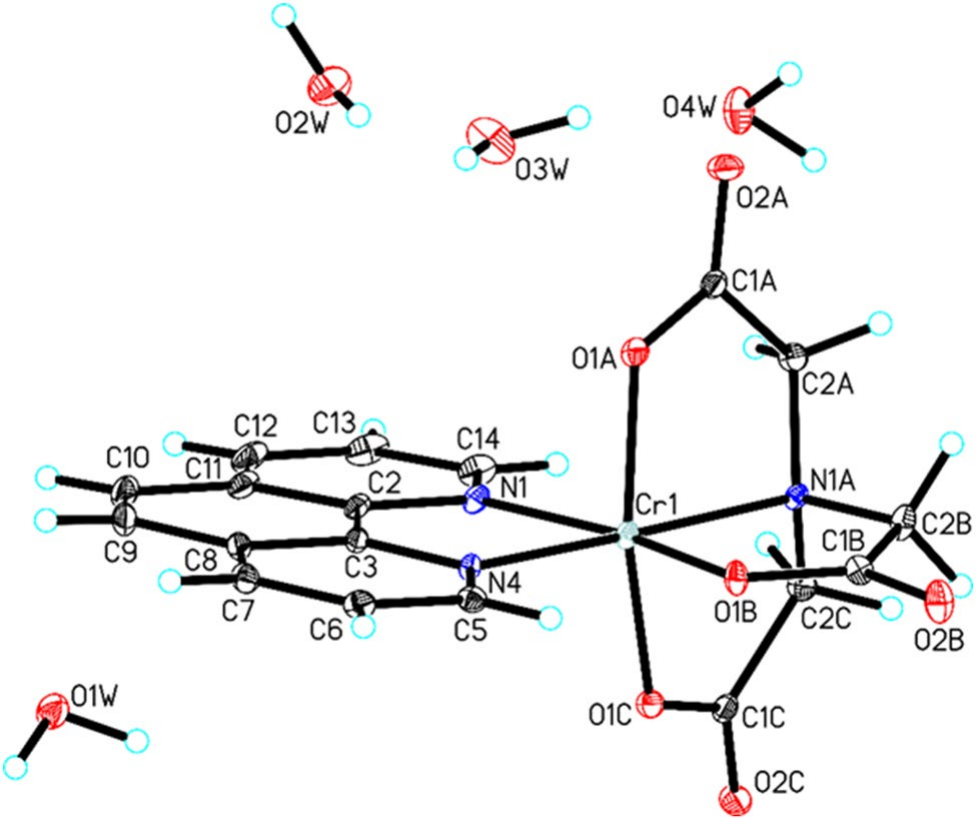
Molecular structure of title compound, showing the atom-labelling scheme (hydrogen bonds are represented by dashed lines).

Cat-CrNP has been tested and characterized towards its physicochemical properties by multi analytical methods such as XRD, elemental analysis, IR, MALDI-TOF–MS, potentiometric conductometric titrations, thermal analysis and scanning electron microscopy. Cat-CrNP is new nitrilotriacetate complex compound of chromium(III) with 1,10-phenanthroline (Fig. 1).

**Figure 2:**
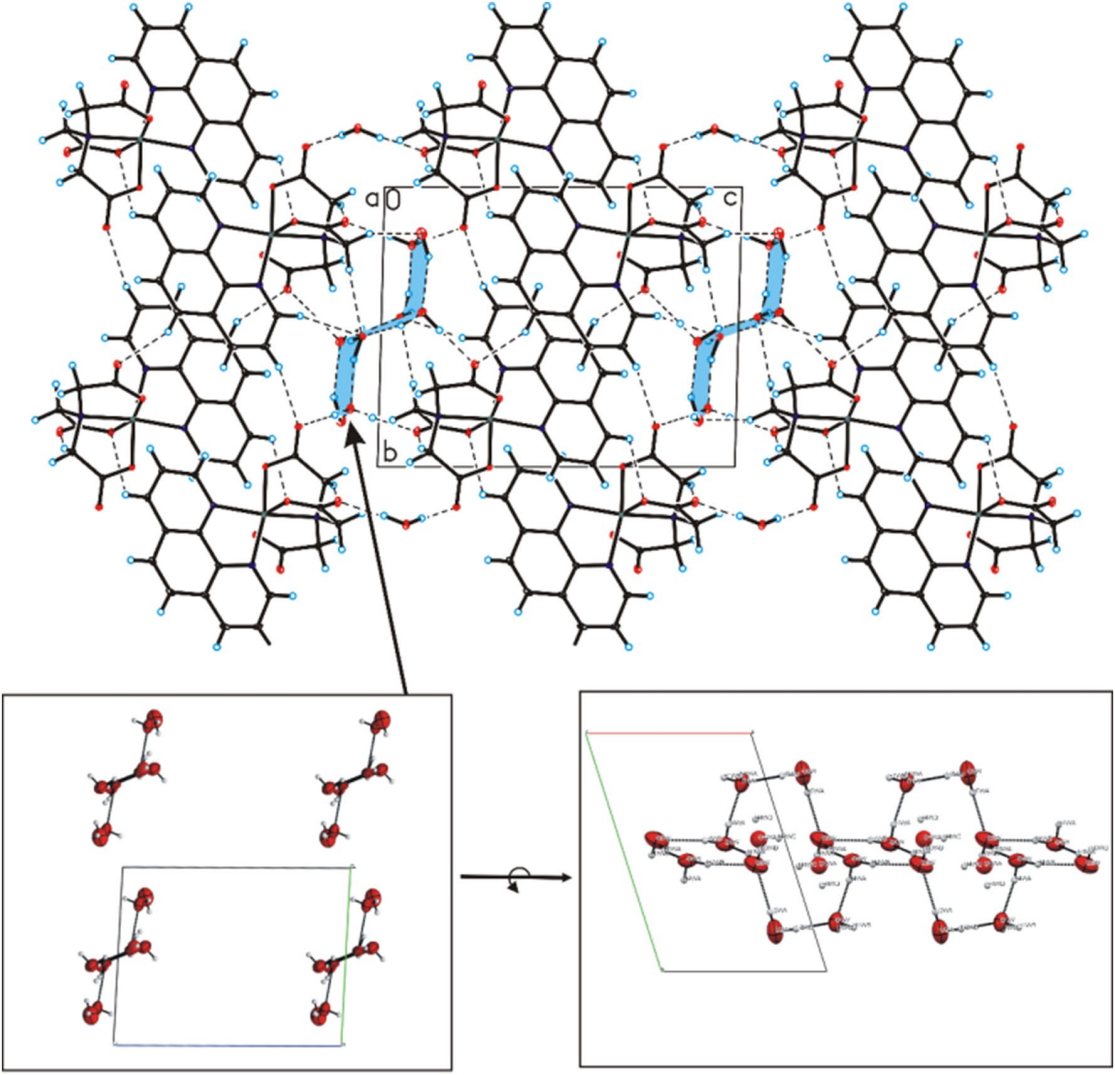
Crystal packing of (nitrilotriacetato-1,10-phenanthroline)chromium(III) tetrahydrate viewed along *a*-axis.

The title compound has an interesting structure. Cat-CrNP was obtained as violet-gold crystals. In the crystal of title compound, molecules of nitrilotriacetate complex compound of chromium(III) with 1,10-phenanthroline are linked via C–H···O hydrogen bonds and π-π interactions to produce blocks along b-axis, whereas water molecules interact via O_(water)_–H···O_(water)_ interactions to form a centrosymmetric, alternately arranged (H_2_O)_4_ (cyclic-planar, tetrameric 0–4-A structure) and (H_2_O)_8_ (cyclic-chair, octameric 0–8-I structure) water clusters, which produce tapes extending along a-axis^30–32^. In the crystal packing, the adjacent blocks and tapes are connected through C–H···O_(water)_ and O_(water)_–H···O hydrogen bonds to form a 3D framework structure (Fig. 2).

Elemental analysis of as-prepared Cat-CrNP revealed that it is composed of C (44.18%), H (4.51%) and N (8.54%). Anal. Calcd.: C, 43.91%, H, 4.51%, N, 8.53%. Thus, experimentally determined composition of the chromium(III) complex compound perfectly fits theoretical findings.

The IR analysis showed that medium intensity stretch band from carboxylic acid occurs at 3440 cm^−1^. In the range 2970–2939 cm^−1^ O–H stretching vibrations of crystallization water are observed. The range 1685–1664 cm^−1^ confirms the presence of C = O stretching of the carbonyl group. The peak at 445 cm^−1^ corresponds to deformation vibrations Cr–N.

MALDI-TOF–MS revealed that Cat-CrNP fragments into M–COO (the peak at 444,052 m/z) and M–COOH (the peak at 443,037 m/z). The potentiometric titration has been used to investigate the stability of the title complex compound by Hyperquad program. The value of the log*β* for Cat-CrNP equal to 8.97 ± 0.05. The value of the log*β* = 8.97 ± 0.05 refers to the final complex compound containing the two ligands nitrilotriacetate anion and 1,10-phenanthroline. The fit of the experimental data to the mathematical model is shown in Fig. 4 in Supplementary Information. In this figure, in the legend on the right, you can see that the mathematical model uses an addition to the MLR system which corresponds to the final complex as M is Cr(III), L is a nitrilotriacetate anion and R is 1,10-phenanthroline. Fig. 4 in Supplementary Information shows that the fit of the experimental values to the theoretical model is very good. The conductometric analysis allow to confirm the composition of the chromium(III) complex compound. The decrease in the conductivity of the solution is maintained to $\text{n}_{\text{NaOH}}{:}\text{n}_{{\text{H}_3}\text{NTA}}=4$, then $\text{n}_{\text{NaOH}}{:}\text{n}_{{\text{H}_3}\text{NTA}}>4$ there is an increase in conductivity. At $\text{n}_{\text{NaOH}}{:}\text{n}_{{\text{H}_3}\text{NTA}}$ equal to 3 the conductivity of the solution is lowered due to the formation of the complex compound.

**Figure 3:**
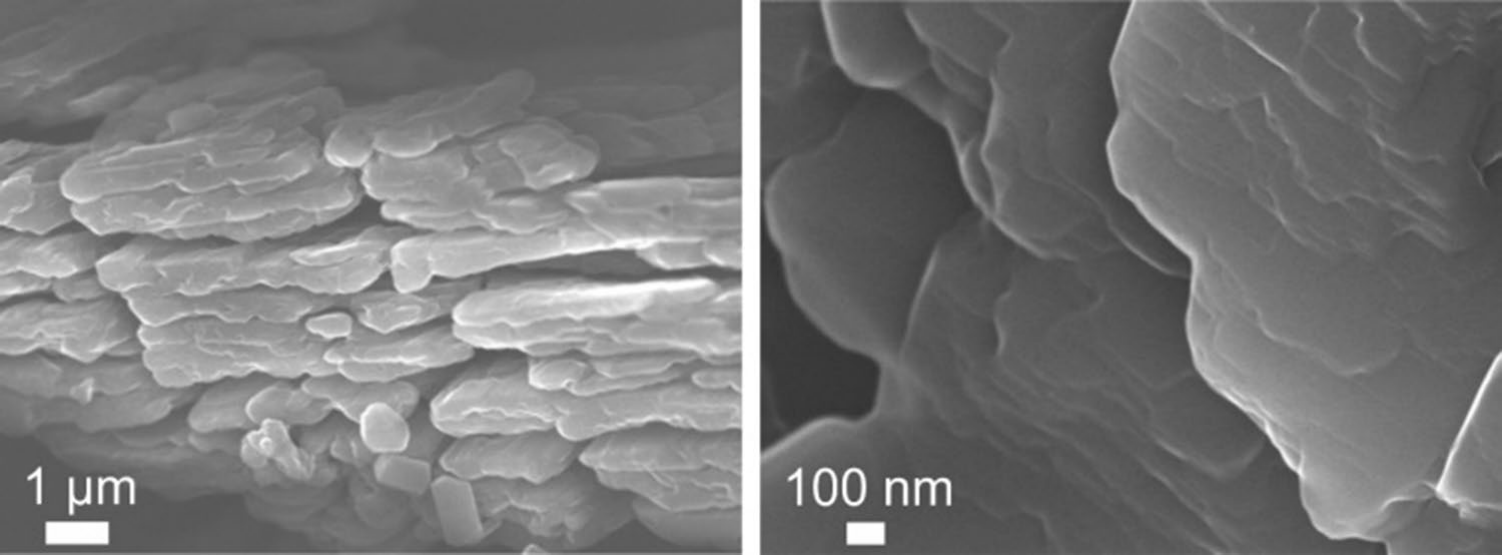
SEM images of Cat-CrNP complex.

Cat-CrNP was subjected to thermal analysis using the thermogravimetric method. The analysis of the thermal decomposition of the coordination compound was carried out in the temperature range from 0 °C to 1000 °C. Cat-CrNP undergoes thermal decomposition in 7 stages. Cat-CrNP is a tetrahydrate therefore TG/DTA analyses shows the thermal weight loss corresponding to the desorption of water at low temperature region. The first stage of decomposition reached the temperature of 80 °C, where the weight loss was 8.23%. In the first stage of thermal analysis, a peak is observed at 62.2 °C, where there was a weight loss of 2.68%. The second stage of thermal decomposition occurred in the temperature range of 80 °C to 140 °C, where a second peak is observed, weight loss (1.23%) at 90.8 °C. The third stage of thermal analysis of Cat-CrNP occurs in the temperature range of 140–380 °C. The weight of the sample during the analysis in this temperature range decreased by 24.65%. In further analysis of TG up to 440 °C a mass loss equal to 10.67% and a peak at 437.7 °C is observed, where 2.38% of the sample mass has been decomposed. Then the sample decomposed in 10.67% to the temperature of 460 °C and in the next stage by 35.94% to 660 °C. In the last stage of decomposition of the sample, the weight loss was 1.53%. Finally, SEM analysis was used for catalysts morphology imaging. As it was shown in Fig. 3, the chromium complex exhibited a plate-like structure with regular edges and smooth surfaces.

Cat-CrNP has been used for the oligomerization of 2-chloro-2-propen-1-ol and ethylene. The oligomers obtained in the oligomerization of 2-chloro-2-propen-1-ol have been characterized by IR. Medium intensity stretch band of the O–H group in the oligomer chain participating in hydrogen bonding occurs at 3475 cm^−1^. CH_2_ bending vibration in the oligomer chain is confirmed by peaks in the range 1457–1402 cm^−1^. C–O stretching vibration occurs at 1178 cm^−1^. C–Cl bending vibrations in the oligomer chain have been confirmed by the presence of the peak at 575 cm^−1^. MALDI-TOF–MS analysis showed that the oligomer sample contains from 4 to 7 mers in the chain. Moreover, the oligomers observed in MALDI-TOF-MS contain precatalyst. An appropriate isotope pattern for Cr^52^ and Cr^53^ which is observed on figures attached in Electronic Supplementary Information i.e. Figure 2 (MALDI-TOF-MS spectrum of Cat-CrNP) and also on Fig. 7 and 11 confirms it. In the case of MALDI-TOF-MS spectra of ethylene and 2-chloro-2-propen-1-ol oligomers the peaks 459 m/z and 460 m/z confirm this. The oligomerization product of 2-chloro-2-propenol was tested for thermal stability. The sample was decomposed in 5 stages. The first stage of decomposition of the sample resulted in a weight loss of 10.21% and it was recorded up to 140 °C. A peak was recorded in the above-mentioned temperature range at a temperature of 101.1 °C, where the weight loss was 1.18%. The second stage of thermal decomposition of the tested sample resulted in a weight loss of 5.91% in the temperature range of 140–200 °C. In the third stage 24.62% of the sample decomposed in in the temperature range of 200–440 °C. In this interval, a peak was recorded at 236.0 °C (weight loss 1.49%). In the penultimate stage, the sample decomposed in 12.01% (440–750 °C), while in the last stage it was 2.37% (750–1000 °C).

The analysis of samples obtained as a result of ethylene oligomerization confirmed the structure of the product obtained. IR spectrum allow to concluded that peaks at 2924 cm^−1^ confirms strong asymmetric CH_2_ stretching vibration in the chain of the oligomer. The peak at 2853 cm^−1^ correspond with strong symmetrical CH_2_ stretching vibration. The presence of strong deformations of CH_2_ in the oligomer chain is confirmed by the peak at 1514 cm^−1^. Peaks in the range 1468–1426 cm^−1^ allow to conclude that weak symmetrical deformation of the terminal −CH_3_ occurs in the oligomer chain. The peak at 857 cm^−1^ confirms the presence of −CH_2_ rocking vibration. MALDI-TOF-MS showed that the product of ethylene oligomerization contains from 12 to 24 mers. In order to understand thermal stability of the obtained oligomers, the sample of oligomers was subjected to the thermal analysis. The sample decomposes in 5 steps. In the first stage of 5.17% undergoes decomposition to 100 °C. Additionally a peak at 83.6 °C was recorded, where the weight loss was 1.23%. The second step of thermal analysis (100–320 °C) resulted in 27.83% weight loss of the sample. The peak at 149.5 °C was recorded, where the sample weight decreased by 2.15%. In the next stage of thermal analysis (320–520 °C) the sample decomposed by 9.38%. The penultimate stage of the sample decomposition was recorded in the temperature range of 520–740 °C (weight loss 6.68%). In the last stage of sample decomposition, the sample decreased by 7.08%.

Glass transition temperatures were determined for the products of oligomerization of 2-chloro-2-propen-1-ol and ethylene by DSC method. In the case of a sample of the 2-chloro-2-propen-1-ol oligomerization product, the cooling curve records the thermal change (Tg) at − 113 °C, and in the case of the heating curve at T =  − 95 °C. In the case of the ethylene oligomerization product sample, the cooling curve records the thermal change (Tg) at − 109 °C, and in the case of the heating curve at T =  − 91 °C.

**Figure 4:**
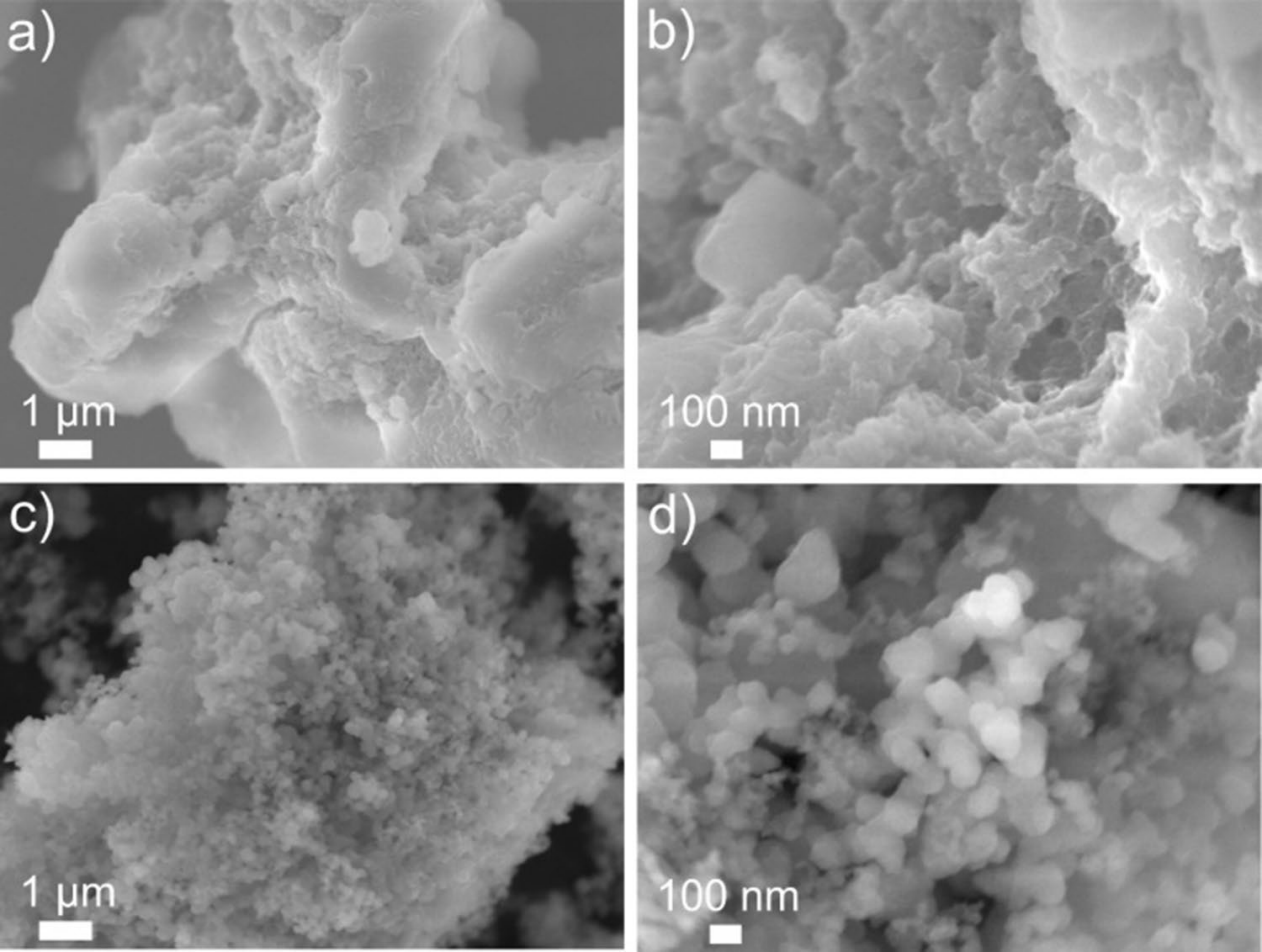
Low and high magnification SEM images of (a, b) 2-chloro-2-propen-1-ol, and (c, d) ethylene oligomers.

The morphology of the 2-chloro 2-propen 1-ol and ethylene oligomers were investigated by SEM, and the results are shown in Fig. 4. SEM images show that oligomers presented small, highly coagulated, and nonuniform particles. According to the literature^33,34^, such a one-dimensional structure might physically provide a better dispersion in the monomers and better exposure of active sites to the reactants thus achieved high catalytic activity^33,34^.

**Figure 5:**
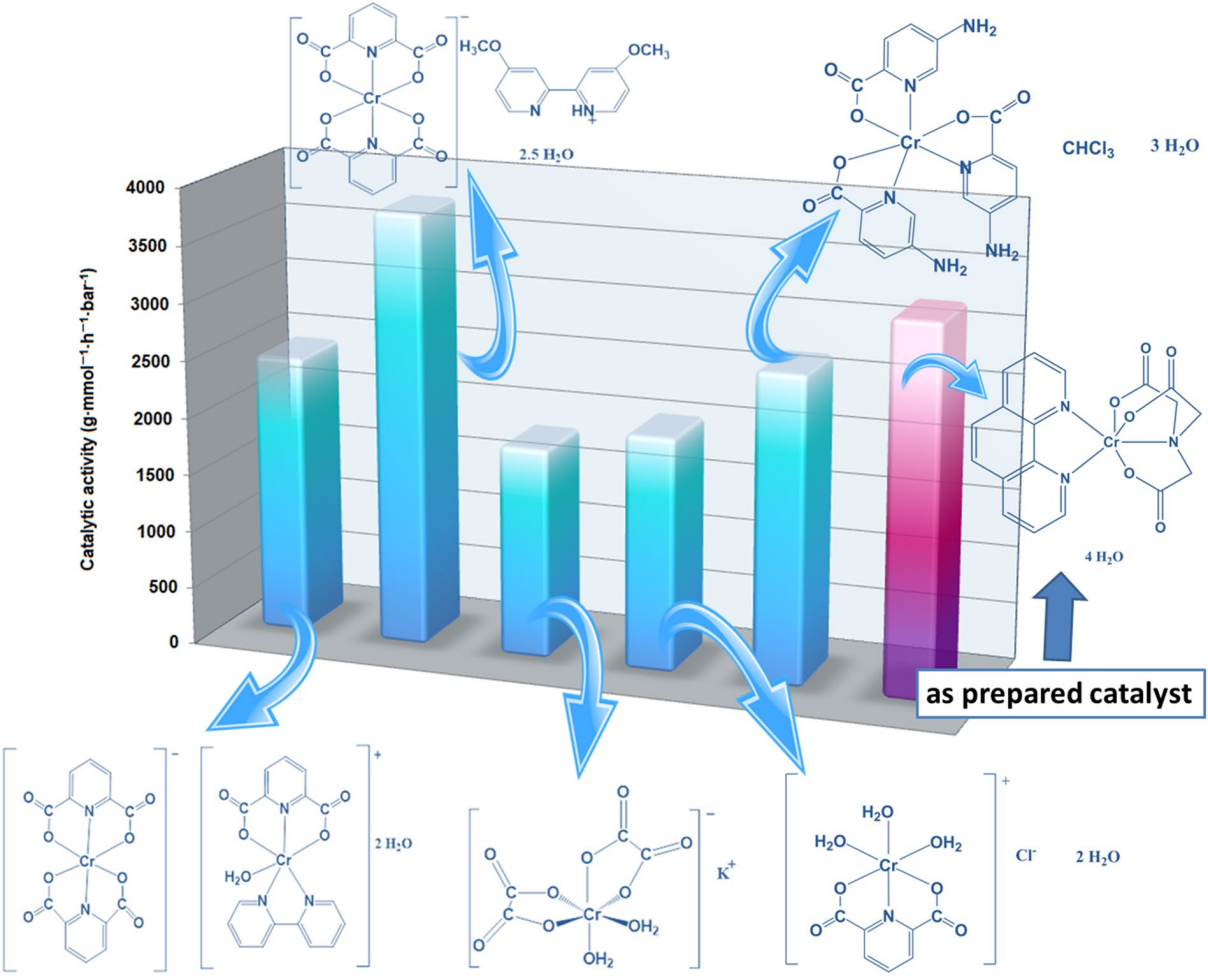
The catalytic activities of Cr(III) complexes for ethylene oligomerization based on^35^.

**Figure 6:**
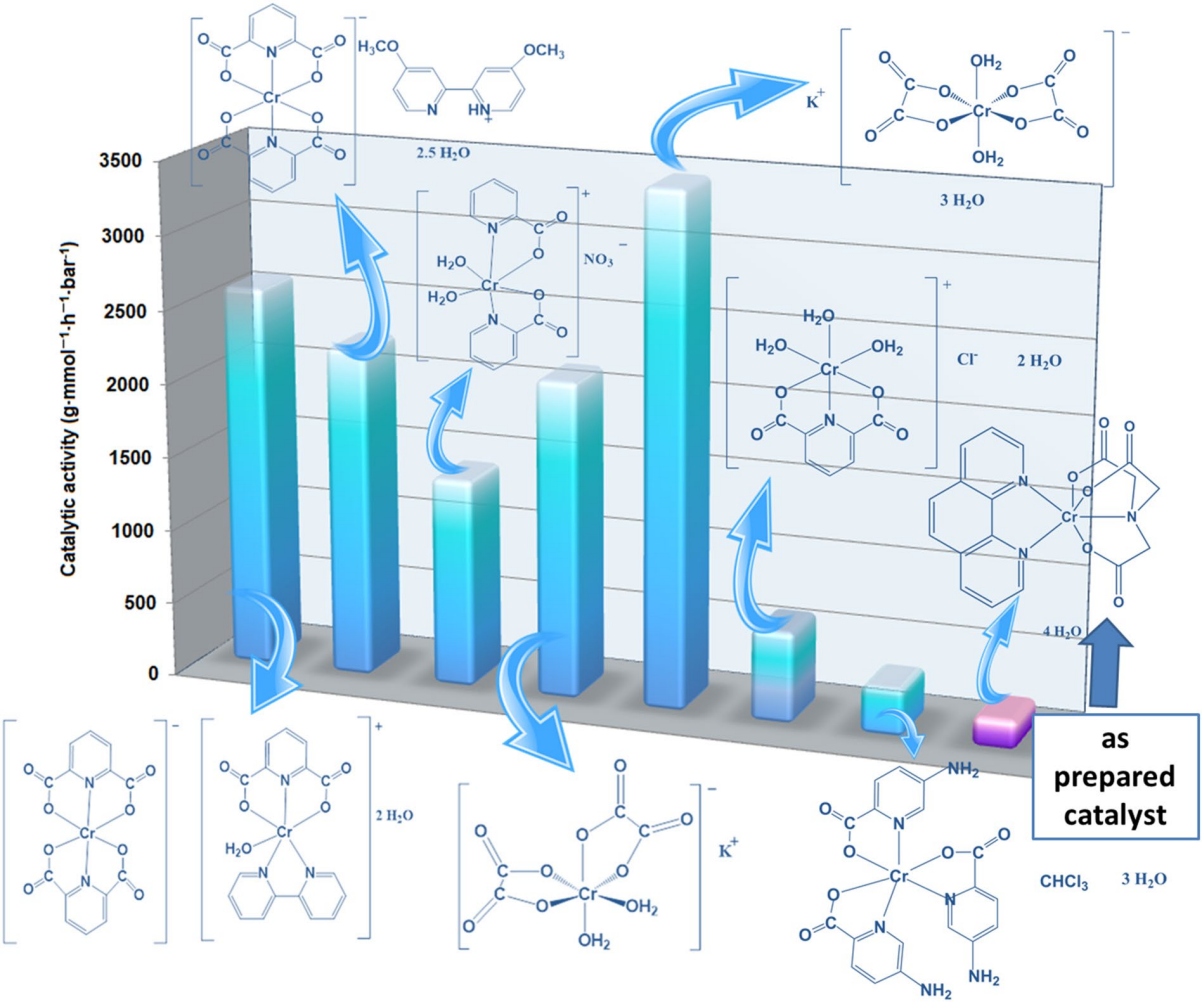
The catalytic activities of Cr(III) complexes for 2-chloro-2-propen-1-ol oligomerization based on^36–40^.

The yield of catalytic oligomerization over Cat-CrNP equaled to 213.92 g · mmol^−1^ · h^−1^· bar^−1^ and 3232 g · mmol^−1^ · h^−1^ · bar^−1^ for the 2-chloro-2-propen-1-ol and ethylene, respectively. It means that Cat-CrNP is very highly active catalyst for the ethylene oligomerization and it is highly active catalyst for the 2-chloro-2-propen-1-ol oligomerization (Figs. 5 and 6). Analyzing other chromium(III) complexes with catalytic properties in ethylene oligomerization (Fig. 5), it can be concluded that the Cat-CrNP—new catalytic material shows catalytic activity similar to the best precatalysts. Catalytic oligomerization of ethylene over Cat-CrNPs is only a slightly lower than that previously reported for [Cr(dipic)_2_]Hdmbipy·2.5 H_2_O (3232 and 3798 g·mmol^−1^·h^−1^·bar^−1^, respectively), nonetheless, synthesis of material reported in this work does not require the usage of thrichlormethane and methanol, and precipitation time was shortened from 2 months to 14 days. It should also be noted that the investigated oligomerization processes with the participation of the new material Cat-CrNP proceed in very mild conditions. Although other catalysts with higher catalytic activity values than the catalyst described in this work are known in the literature, but synthesis of Cat-CrNP is very simple, cheap and it crystallizes very quickly, comparing to synthesis procedure of commercially available catalysts. In addition, the synthesis uses mainly an environmentally friendly solvent–water.

Comparing the catalytic activity of the new precatalyst described in this work with the precatalysts used for the oligomerization of ethylene and being transition metal complex compounds with N^N bidentate ligands, it can be concluded that Cat-CrNP exhibits outstanding catalytic activity. Iron(II) complex compounds with N^N bidentate ligands, such as diimine (Ph_2_C = N)_2_C_2_H_4_ ligand show low catalytic activity in the range from 100 to 550 g·mmol^−1^·h^−1^·bar^−1^ ^41^. Likewise nickel(II) complexes with N^N bidentate ligands exhibit lower catalytic activity than Cat-CrNP. These nickel(II) complex compounds exhibit a maximum catalytic activity of 1638 g·mmol^−1^·h^−1^·bar^−1^ in ethylene oligomerization^42^.

## Conclusions

This application brings a new concept of using a new coordination compound obtained in a crystalline form as a material with catalytic properties in the oligomerization of ethylene and 2-chloro-2-propen-1-ol. Physicochemical and catalytic properties of Cat-CrNP were fully described in this article. Cat-CrNP crystallizes in the triclinic system. It is thermally decomposed in 7 stages in the range 0 °C to 1000 °C. While in the MALDI-TOF–MS analysis it fragmented into M minus COO and M minus COOH. Cat-CrNP has high catalytic activity for 2-chloro-2-propen-1-ol and ethylene oligomerizations. The sample of the oligomerization products contains from 4 to 7 mers and from 12 to 24 mers for 2-chloro-2-propen-1-ol and ethylene oligomerizations, respectively. The results described in this article create the prospect of using the new material, Cat-CrNP, as a catalyst in the oligomerization and polymerization reactions of various olefins.

## Methods

### Synthetic procedures

#### Cat-CrNP synthesis

To a suspension of nitrilotriacetic acid (0.57 g in 40 mL H_2_O) was added an aqueous potassium hydroxide solution (0.51 g in 10 mL H_2_O) to convert the acid to a highly water soluble salt. An aqueous CrCl_3_ solution (0.54 g in 10 mL of water) was added to the reaction solution. An ethanolic solution of 1,10-phenatroline (0.54 g in 25 mL of ethanol) was then added. The entire process was carried out using a magnetic stirrer, and in the last step, the reaction solution was heated under reflux at a temperature of 50 °C. Cat-CrNP crystallizes within 14 days. The molar mass of Cat-CrNP is equal to 492.38 g·mol^−1^ and the yield of the synthesis of the complexes is 68%.

### 2-Chloro-2-propen-1-ol oligomerization

2-Chloro-2-propen-1-ol was subjected to oligomerization under the following conditions: at room temperature, under atmospheric pressure, and under a nitrogen atmosphere. In the first oligomerization step, Cat-CrNP (3 µmol) was dissolved in toluene (2 mL). Then MMAO-12 (3 mL) was added to the resulting toluene solution. In the last step, 2-chloro-2-propen-1-ol was slowly added. After 25 min of the reaction the gel product was formed. The final product obtained was dried and weighed.

### Ethylene oligomerization

A solution of precatalyst was prepared by dissolving 3 μmol of Cat-CrNP in 2 mL of toluene in a glass tube on the magnetic stirrer. The oligomerization process was carried out at 0.12 bar, at room temperature and nitrogen atmosphere. In the next reaction stage, MMAO-12 (3 mL) solution was introduced. The solution was allowed to mix for a while, then ethylene was added all the time until the process was complete. The gel product was obtained after 15 min of the reaction. Then the formed product was dried and weighed.

### Physico-chemical characterization

Diffraction data were collected on an Oxford Diffraction Gemini R ULTRA Ruby CCD diffractometer (T = 295(2) K, MoK_α_ (λ = 0.71073 Å) radiation, Table 1—see Electronic Supplementary Information) and were reduced using CrysAlis RED software (ver. 1.171.41.16a)^43^. The structures were refined and solved using the SHELX package (ver. 2017/1)^44^. The water molecule including O4W atom has orientation disorders with refined site-occupancy factors equal 0.50(7). H-atoms from water molecules were located on a difference Fourier map and refined with restraints (DFIX command) with *d*_*(*O–H)_ = 0.95 Å and U_iso_(H) = 1.5U_eq_(O), while H–atoms bound to C–atoms were placed geometrically and refined using a riding model with *d*_(C–H)_ = 0.93–0.97 Å and U_iso_(H) = 1.2U_eq_(C). All interactions were identified using the PLATON program (ver. 181,115)^45^.

The ORTEPII, PLUTO-78 and Mercury (ver. 2020.2.0) programs were used to prepare the molecular graphics^46–48^. A disordered part of the O4W water molecule was omitted from the figures, for clarity. Full crystallographic details for title compound have been deposited in the Cambridge Crystallographic Data Center (deposition No. CCDC 2,058,007) and they may be obtained from www: http://www.ccdc.cam.ac.uk, e-mail: deposit@ccdc.cam.ac.uk or The Director, CCDC, 12 Union Road, Cambridge, CB2 1EZ, UK.

The elemental analysis of Cat-CrNP was examined by performed on the Vario EL analyzer Cube (CHNS). MALDI-TOF–MS was carried out for Cat-CrNP and for the oligomerization products using the BrukerBiflex III spectrometer. 2,5-Dihydroxybenzoic acid was the matrix used during the recording of spectra. IR spectra were recorded ranging from 4000 to 600 cm^−1^ in KBr pastil (BRUKER IFS 66 spectrophotometer). Potentiometric titration was performed using the CerkoLab system (T = 298.15 K ± 0.10 K Lauda E100 circulation thermostat), Hamilton syringes (5 mL), a measuring cell and a combined pH electrode Schott Blue Line 16. Calibration of the electrode was carried out the weighing method. Titration systems were follows: (1) H_3_NTA (2 mM), (2) Cr^3+^ (2 mM) + H_3_NTA (2 mM), (3) Cr^3+^ (2 mM) + H_3_NTA (2 mM) + phen (2 mM), where H_3_NTA–nitrilotriacetic acid, phen–1,10-phenantroline. A standard solution of NaOH (50 mM) was used as the titrant. The titrant was added every 60 s in an amount of 0.020 mL. The each measurement consisted of 300 steps. Conductometric titration (CerkoLab equipment, a magnetic stirrer, a 5 ml Hamilton’s syringe) was carried out with a conductometric cell (*k* = 0.096 cm^−1^ HYDROMET) at 298.15 K ± 0.10 K (E100 Lauda circulation thermostat). The conductivity probe was calibrated using KCl solutions with conductivity of 84 µS and 200 µS purchased commercially from the Hamilton. 50 mM NaOH was applied as the titrant. The following systems were titrated: 1) H_3_NTA (2 mM), 2) Cr^3+^ (2 mM) + H_3_NTA (2 mM), 3) Cr^3+^ (2 mM) + H_3_NTA (2 mM) + phen (2 mM). Thermal analysis (TG) was performed on NETZSCH TG 209 instrument in the temperature range from 0 to 1000 °C in an argon atmosphere. The mass of samples subjected to thermal analysis was about 5 mg. The morphologies of the as-prepared samples were investigated using field-emission SEM using JSM-7610F, JEOL. The differential scanning calorimetry (DSC) studies were performed using the equipment from Mettler Toledo in the range from − 150 to 500 °C with the heating rate of 10 °C/min in the inert atmosphere. The sample to the DSC measurements was about 5 mg. The calibration was carried out based on standards for thermal analysis (ind, n-octane). The liquid nitrogen was used to cooling.

## Supplementary Information

Supplementary Information.
