# Supplementary material for: Cat-CrNP as new material with catalytic properties for 2-chloro-2-propen-1-ol and ethylene oligomerizations
Source: Sci Rep. 2021 Jul 26;11:15212. doi: 10.1038/s41598-021-94056-0 (PMC8313536; doi:10.1038/s41598-021-94056-0)
Supplement: Supplementary file 1 — Supplementary Information. [file 41598_2021_94056_MOESM1_ESM.doc]

**Electronic Supplementary Information**

**Cat-CrNP as new material with catalytic properties for 2-chloro-2-propen-1-ol and ethylene oligomerizations**

Jacek Malinowski ^a^, Dagmara Jacewicz ^a^, Artur Sikorski ^a^, Mariusz Urbaniak ^b^, Przemysław Rybiński ^b^, Patrycja Parnicka ^a^, Adriana Zaleska-Medynska^a^, Barbara Gawdzik*^b^ and Joanna Drzeżdżon^a^

^a^ Faculty of Chemistry, University of Gdansk, Wita Stwosza 63, 80-308 Gdansk, Poland

^b^ Institute of Chemistry, Jan Kochanowski University, Swietokrzyska 15 G, 25-406 Kielce, Poland

**Table of contents**

1. **Structure of Cat-CrNP …………………………………………………………….3**
2. **The studies of 2-chloro-2-propen-1-ol oligomerization…………………………...7**
3. **The studies of ethylene oligomerization …………………………………………..9**

**1. Structure of Cat-CrNP**

**Table 1**. Crystal data and structure refinement for title compound.

| Chemical formula | CrC_18_H_22_N_3_O_10_ |
| --- | --- |
| Formula weight/g·mol^-1^ | 492.38 |
| Crystal system | triclinic |
| Space group | P-1 |
| *a*/Å | 7.4110(6) |
| *b*/Å | 11.0109(7) |
| *c*/Å | 13.6138(11) |
| *α*/° | 88.285(6) |
| *β*/° | 79.705(7) |
| *γ*/° | 72.291(7) |
| *V*/Å^3^ | 1040.9(2) |
| *Z* | 2 |
| *T*/K | 293(2) |
| *λ*_Mo_/Å | 0.71073 |
| *ρ_cal_*_c_/g·cm^–3^ | 1.571 |
| *F(000)* | 476 |
| µ/mm^-1^ | 0.612 |
| *θ* range/° | 3.54-25.00 |
| Completness *θ* /% | 99.5 |
| Reflections collected | 6785 |
| Reflections unique | 3652 [R_int_= 0.0696] |
| Data/restraints/parameters | 3652/10/324 |
| Goodness of fit on *F^2^* | 0.995 |
| Final R_1_ value (*I*>2σ(*I*)) | 0.0668 |
| Final *w*R_2_ value (*I*>2σ(*I*)) | 0.0899 |
| Final R_1_ value (all data) | 0.1256 |
| Final *w*R_2_ value (all data) | 0.1092 |
| CCDC number | CCDC 2058007 |

***Table 2****. Hydrogen bonds geometry for title compound.*

| **D–H···A** | **d(D–H) [Å]** | **d(H···A) [Å]** | **d(D⋯A) (Å)** | **∠D–H⋯A (°)** |
| --- | --- | --- | --- | --- |
| O1W–H1WA···O2A^i^ | 0.92(2) | 1.81(3) | 2.722(5) | 175(6) |
| O1W–H1WB···O2B^ii^ | 0.94(2) | 1.81(4) | 2.732(6) | 169(4) |
| O2W–H2WA···O3W | 0.94(2) | 2.18(4) | 3.070(6) | 158(6) |
| O2W–H2WB···O1W^iii^ | 0.93(2) | 1.97(4) | 2.845(6) | 157(6) |
| O3W–H3WA···O4W | 0.95(2) | 1.70(5) | 2.614(7) | 159(6) |
| O3W–H3WB···O2C^iv^ | 0.94(2) | 1.90(5) | 2.830(7) | 174(6) |
| O4W–H4WA···O1W^v^ | 0.94(2) | 1.95(5) | 2.864(7) | 163(8) |
| O4W–H4WB···O3W^vi^ | 0.95(2) | 2.14(5) | 3.047(7) | 162(8) |
| C6–H6A···O1A^vii^ | 0.93 | 2.58 | 3.098(6) | 115 |
| C7–H7A···O1A^vii^ | 0.93 | 2.56 | 3.089(6) | 117 |
| C10–H10A···O2C^viii^ | 0.93 | 2.49 | 3.338(7) | 152 |
| C2B–H2BA···O2A^ix^ | 0.97 | 2.55 | 3.489(6) | 164 |
| C2A–H2AA···O4W^vi^ | 0.97 | 2.55 | 3.511(8) | 169 |
| C13–H13A···O2B^x^ | 0.93 | 2.36 | 3.261(6) | 164 |
| C2A–H2AB···O2W^vi^ | 0.97 | 2.57 | 3.370(7) | 140 |
| C14–H14A···O4W^vi^ | 0.93 | 2.45 | 2.273(9) | 147 |
| Symmetry code: (i) x,y,1+z; (ii) 1-x,2-y,1-z; (iii)-x,1-y,1-z; (iv)-1+x,y,z; (v) x,y,-1+z; (vi) 1-x,1-y,-z; (vii) -x,2-y,1-z; (viii) 1-x,1-y,1-z; (ix) 1+x,y,z; (x) x,-1+y,z. | | | | |

**Figure 1.** IR spectrum of Cat-CrNP.

**Figure 2.** MALDI-TOF-MS spectrum of Cat-CrNP.

**Figure 3.** Results of TG analysis of Cat-CrNP.


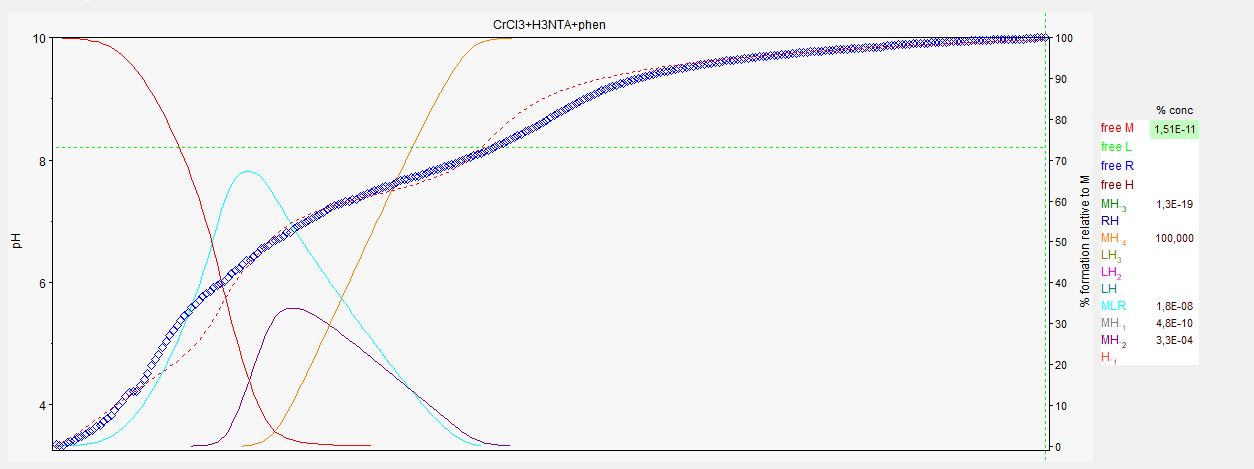


**Figure 4.** Results of potentiometric titration of the system Cr(III):H_3_NTA:phen.

**
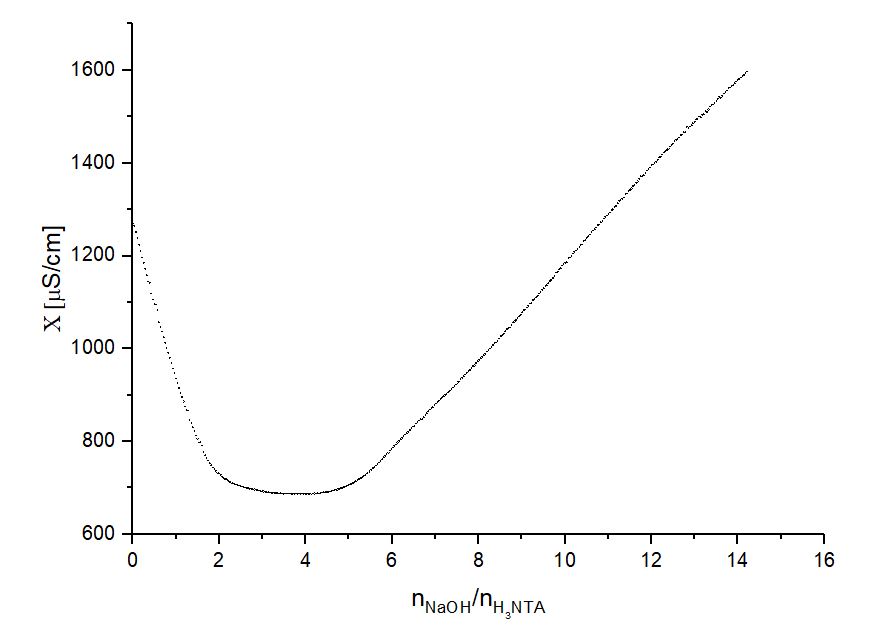
**

**Figure 5.** Results of conductometric titration of the system Cr(III):H_3_NTA:phen.

**2. The studies of the 2-chloro-2-propen-1-ol oligomerization**

**Figure 6.** IR spectrum of oligomers.

**Figure 7.** MALDI-TOF-MS spectrum of oligomers.

**Figure 8.** Results of TG analysis of oligomers.


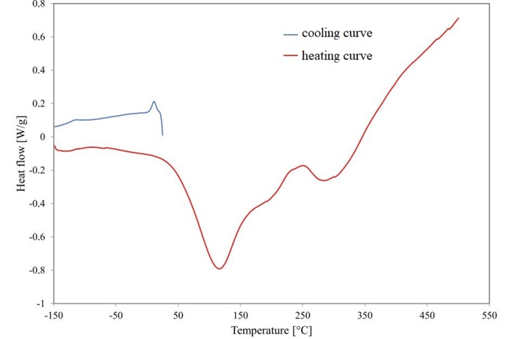


**Figure 9.** The DSC results for sample of the 2-chloro-2-propen-1-ol oligomerization product.

**3. The studies of the ethylene oligomerization**

**Figure 10.** IR spectrum of oligomers.

**Figure 11.** MALDI-TOF-MS spectrum of oligomers.

**Figure 12.** Results of TG analysis of oligomers.

**
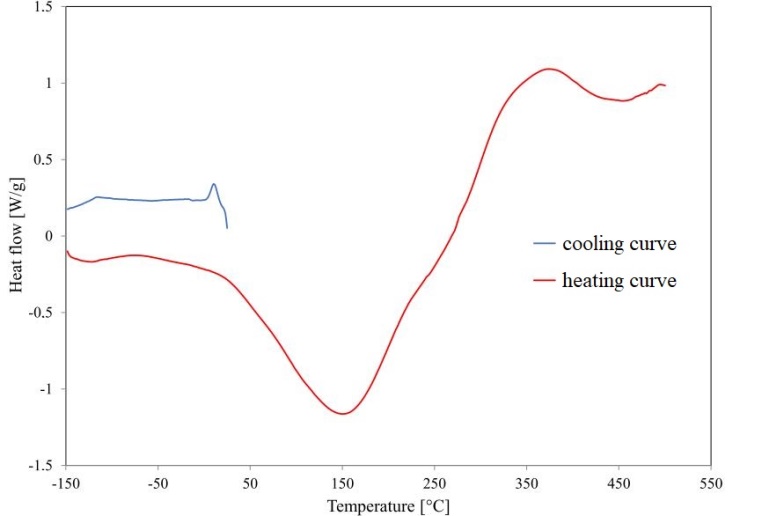
**

**Figure 13.** The DSC results for sample of the ethylene oligomerization product.
